# Melanoma: A model for testing new agents in combination therapies

**DOI:** 10.1186/1479-5876-8-38

**Published:** 2010-04-20

**Authors:** Paolo A Ascierto, Howard Z Streicher, Mario Sznol

**Affiliations:** 1 Unit of Medical Oncology and Innovative Therapy, National Tumor Institute, Naples, Italy; 2 Cancer Therapy Evaluation Program, National Cancer Institute, Bethesda, MD, USA; 3 Melanoma Program, Yale University School of Medicine, New Haven, CT, USA

## Abstract

Treatment for both early and advanced melanoma has changed little since the introduction of interferon and IL-2 in the early 1990s. Recent data from trials testing targeted agents or immune modulators suggest the promise of new strategies to treat patients with advanced melanoma. These include a new generation of B-RAF inhibitors with greater selectivity for the mutant protein, c-Kit inhibitors, anti-angiogenesis agents, the immune modulators anti-CTLA4, anti-PD-1, and anti-CD40, and adoptive cellular therapies. The high success rate of mutant B-RAF and c-Kit inhibitors relies on the selection of patients with corresponding mutations. However, although response rates with small molecule inhibitors are high, most are not durable. Moreover, for a large subset of patients, reliable predictive biomarkers especially for immunologic modulators have not yet been identified. Progress may also depend on identifying additional molecular targets, which in turn depends upon a better understanding of the mechanisms leading to response or resistance. More challenging but equally important will be understanding how to optimize the treatment of individual patients using these active agents sequentially or in combination with each other, with other experimental treatment, or with traditional anticancer modalities such as chemotherapy, radiation, or surgery. Compared to the standard approach of developing new single agents for licensing in advanced disease, the identification and validation of patient specific and multi-modality treatments will require increased involvement by several stakeholders in designing trials aimed at identifying, even in early stages of drug development, the most effective way to use molecularly guided approaches to treat tumors as they evolve over time.

## Current prospects for melanoma therapy

The only approved chemotherapy for metastatic melanoma, DTIC, or its oral equivalent temozolomide, has a response rate of about 10% and a median survival of 8-9 months. The other approved agent for advanced melanoma is high dose interleukin-2, which can induce dramatic complete and durable responses. However, only one patient in twenty derives lasting benefit. Multi-agent combinations [1-7] and bio-chemotherapy regimens [8-15] were reported to produce much higher objective response rates in phase 2 trials, but did not improve overall survival.

A series of scientific and clinical advances in the past decade has led to a rapid evolution of new treatment strategies. Mutations in B-RAF and c-Kit, have recently been proven to be therapeutic targets in phase 1 clinical trials [16]. Of great potential, a small molecule inhibitor of B-RAF, PLX 4032, induced tumor regression in 70% of cases with a 9 month median progression free survival in the 70% of patients whose metastatic tumors express a specific mutant of B-RAF (V600E). Substantial attention has been focused on the biological mechanisms that led to the success of PLX4032, as other single agent B-RAF and downstream MEK inhibitors were less active [17-19].

Successful results were also reported for imatinib (another kinase inhibitor) in a small but distinct subset of patients with c-kit mutated tumors [20]. Finding additional relevant treatment targets will likely extend the number of patients for whom highly active initial treatment regimens may be chosen.

With regard to biological therapies, the combination of a chemotherapy preparative regimen with adoptive T-cell immunotherapy [21], while technically demanding, has a high response rate, demonstrating the potential efficacy of activated T cells. In addition, the removal of immunologic inhibition at checkpoints in T-cell activation and effector function by agents such as anti-CTLA4 antibody [22,23] results in tumor regression. These approaches may be even more active when combined with other agents that activate or inhibit key molecular regulators of T-cell function [24]. It may be possible to increase the durability of cell signaling agents and enhance the effects of immune-mediated responses if the best way to combine the distinct advantages of each could be identified. Although only a subset of patients achieve durable remissions follow the administration of single biological agents, it been not yet been possible to predict a responsive subgroup to guide patient selection. However, specific activating mutations required for cell signaling inhibitors should not be a limitation. Thus, patient selection for driver mutations, emergence of resistance, timing and durability of responses, and the need for chronic therapy are different but potentially complementary for each modality. It is presently not known whether responders to immunotherapy overlap with responders to targeted agents, but it is likely that most patients would benefit from a combinatorial approaches in which various agents are given together or in sequence.

Combination chemotherapy for cancer was established in the 1960s when the treatment of acute lymphocytic leukemia and lymphoma followed the strategy of antibiotic therapy for tuberculosis in which two or more drugs, each with a different mechanism of action were most effective. In principle, the agents used in the combinations should have additive effects on tumor growth and non-overlapping toxicity. Individual agents used in combination have generally been tested in phase 1 trials to determine the maximal tolerated dose (MTD) and in phase 2 studies at the highest tolerated dose to determine activity based on objective response rates. This sequential approach to drug development is well established, but may not be sufficient to effectively test new promising agents in combination in early phases of development. We may learn from the experience with HAART (Highly Active Anti-Retroviral Therapy) used to treat patients with HIV infection. The discovery of highly potent single drug treatment has lead to dramatic responses but also to rapid drug resistance. Combinations of three or four different antiretroviral drugs, such as nucleoside and non-nucleoside reverse transcriptase and protease inhibitors, block HIV replication and control viral load, reducing the emergence of HIV escape variants and maintaining CD4+ T cell numbers. Moreover, the recognition that monitoring viral load was a useful surrogate biomarker of viral dynamics, overall treatment efficacy, and survival, allowed drugs to be tested efficiently and more accurately [25,26]. The rapid emergence of a multitude of agents with novel targets and mechanisms of action will require changes in the way combinations are developed. In order to effectively validate and expand targeted therapy, the rationale supporting clinical trials design will need to be adapted to keep pace with the opportunities provided by these new agents. Dropping useful agents in early phase single agent development because they may not induce rapid changes in tumor size might be avoided by monitoring effects on specific targets and tumor growth rates. Finding more accurate predictors of biologic activity and overall survival should improve the accuracy of go-no-go decisions for advancing to phase 2 and phase 3 trials [27-29]. Understanding molecular tumor biology, pharmacodynamic markers, and imaging technology should lead to the development of biomarkers as trial end points that can help develop active regimes more effectively while reducing the number of unsuccessful studies [30,31].

## Early Experience with Molecular Targeted Treatment of Melanoma

The single activating mutation in B-RAF, V600E, is found at all stages of melanoma, including 70% of patients with metastatic melanoma [32-34]. The first attempt to use a B-RAF inhibitor, sorafenib, showed little activity in melanoma, and combinations of sorafenib with chemotherapy were not superior to chemotherapy alone in randomized trials [35-37]. However, in a phase 1 study [16], PLX4032, a B-RAF inhibitor with increased specificity for the V600E mutant protein, has demonstrable activity. As exciting as these early results appear, there were no complete responses reported, a third of patients whose cancer bore the target mutation did not respond, and most patients progressed after 9 months. Several mechanisms may be associated with both primary and secondary resistance even with continued inhibition of B-RAF. Almost all resistant tumor will have inactivating PTEN point mutations, over expression of the PI3K/mTOR/Akt pathway, and in addition, feedback loops stimulating downstream pMEK or a switch to the dominance of C-RAF. These all suggest possible targets to be included in combination therapy [38,39]. It is generally accepted that treatment may need to target both active pathways and it is possible that the combined effects may be measured on a downstream target [40].

To make matters more complex, ATP-competitive RAF kinase inhibitors can have opposing effects depending on the cellular context; growth arrest and apoptosis occur in BRAF^V600E ^tumors, while in the setting of KRAS mutations and wild type B-RAF, inhibitors can activate the RAF-MEK-ERK pathway enhancing tumor growth [41,42].

Continuing the trend in melanoma, another example of patients selection for targeted therapy is c-Kit mutations., which are often present in acral and mucosal tumors. Treatment with c-Kit inhibitors results in a 50% or greater response rate [20]. However, as a caution against over generalizations, the activation of non mutated c-KIT/SCF in uveal melanoma does not translate into clinical efficacy [43]. This suggests that responses are predicted by specific gene mutations and resulting molecular pathogenesis (such as the L576P on exon 11 or the V642E on exon 13) rather than overall protein expression or activation [20]. Common in uveal melanomas, mutations in the G proteins, GNACQ and GNAC11, are more difficult to target than serine kinases and novel biochemical approaches should be sought. New clinical trials based on the discovery of targets, such as the EGFB4 and related activating mutations found in 20% of patients may increasingly broaden the number of patients that could be treated with a first line highly active agent [44].

## Suggestions for combination therapy

Most advanced tumors have developed multiple growth and survival pathways, so that changing their natural history will require multi-potent treatment strategies. Several publications in the past 5 years have suggested new strategies for designing rational treatment combinations. The idea of limiting growth through inhibition of a single pathway, to which the tumor is "addicted", emerged from clinical trials with imatinib in BCR/Abl CML [45], and c-Kit in GIST [46], Trastuzumab in Her-2 neu positive breast cancer [47], and more recently, EGFR in a subgroup of patients with non small cell lung cancer [48]. For these agents resistance often develops through selection of escape mutations in the targeted kinase. Identifying these resistance pathways provides an opportunity to change treatment accordingly [49]. In the case of melanoma with the B-RAF mutation, positive feedback loops may paradoxically lead to over expression of inhibited pathways downstream. Combining inhibition of the PI3K-mTOR pathway with MEK inhibitors may effectively treat KRAS mutated lung cancers, making this combination a high priority that was unexpectedly discovered by RNA screening [50]. Another appealing concept takes advantage of the intrinsic vulnerability of a tumor; for example, combining a DNA repair inhibitor of PARP with a DNA damaging agent may greatly enhance effectiveness in tumors that have already lost one pathway of DNA repair. A striking example is emerging in the treatment of patients with BRCA-1 mutations [51-53].

It is worth noting the increasing value of RNA interference (RNAi) in discovery and functional validation for potential therapeutic targets identified through large-scale RNAi screens. In fact, gene-specific RNAi provides a powerful tool to demonstrate that specific knockdown of a mutant allele triggers cell death or proliferative arrest in oncogene-dependent cell lines. The tumor specific context, for example of PTEN deficiency with PI3K activation, allows more accurate screening of new agents compared to cells lines with intact PTEN [54,55]. RNA screens may provide unexpected findings that suggest therapeutic combinations in resistant disease. For example, ectopic expression of two genes that act on retinoic acid (RA) signalling can cause resistance to growth arrest and apoptosis induced by inhibitors of histone deacetylase (HDACI) of different chemical classes. This suggests that that the RA pathway may be a rate-limiting target of HDACI which could lead to strategies that enhance the therapeutic efficacy of HDACI [56].

Moreover, for planning therapy, it will be important to distinguish driver mutations from passenger mutations, as recent studies have revealed that many tumors (i.e. human colorectal cancers) undergo numerous genetic and epigenetic alterations. These alterations likely derive from a mixture of "drivers" that play a causal role in tumor development and progression, and "passengers" that have little or no effect on tumor growth. The design of targeted therapeutics may be dependent on the ability to distinguish drivers from passengers [57,58].

## Combining signaling inhibitors with other strategies

Sulllivan and Atkins [59] and Palmieri et al. [34] reviewed the use of targeted agents in melanoma and a more general discussion about therapy combinations was provided by Kwak, Clark, and Chabner [60]. Most combinations reported in these reviews involve chemotherapy and often demonstrate how, after safety evaluation in early clinical trials, promising new agents and combinations may stall and never reach the threshold to justify longer, larger, and more expensive trials. Moreover, these trials result in little progress if single agents and empiric combinations are not adequately controlled or analyzed. One advantage of introducing rationally designed combination therapy at an early stage, would be gaining experience in the drug's potential even while single agent development is progressing.

Molecular characterization of the tumor before and during treatment should take a paramount role in trial design particularly when mutations or activated pathways are targeted. Similarly, changes in tumor phenotypes need to be monitored during the natural progression of the disease or in response to the selective pressure exercised by the treatment. Molecular diversity among tumors and within tumors may account for the high degree of variability in response to treatment even in tumor lines with the same primary drivers. Treatment will need to be re-evaluated and changed on a continual basis over time, much as in chronic infections such as for malaria, tuberculosis or HIV. Introducing multiple drugs individually that increase survival by months may not result in prolonged control of the malignant process, but rather in a serial selection of resistant mutant cancer cell populations ever more difficult to control with available therapeutic tools.

Melanoma and renal cell carcinoma have long been held as examples of immunogenic tumors and two kinds of immunotherapy, IFN-alpha in the adjuvant setting and IL-2 for metastatic melanoma, are currently approved [61]. Perhaps the most impressive results in the immunotherapy of melanoma are achieved with adoptive transfer of autologous tumor-reactive lymphocytes which can induce rapid objective responses in up to 70% of recipients, including those with large tumor masses. The activity of adoptively transferred T cells with recombinant or chimeric receptors has strongly supported this approach [62]. In contrast to the dramatic effects of adoptive T-cell therapy, vaccines have shown far less interesting effects. None the less, a phase 3 trial combining IL-2 with a single melanoma antigen epitope has demonstrated a significant improvement in overall response rate, progression free survival, and a strong trend in improvement of overall survival compared to treatment with IL-2 alone [63]. Interestingly, the response rate induced by the administration of peptide alone was minimal, strongly emphasizing the need to test combinations. Results from an ongoing phase 3 study in which vaccination against Mage-3 is combined with a novel adjuvant are anxiously awaited. The study identified a transcriptional signature in tumors that are likely to respond to vaccine suggesting that even for immunotherapy, predictors of responsiveness could be used in the future for patient stratification [64].

Reversing immunologic suppression by intervention with anti-CTLA-4, and more recently anti-CD40, anti-PD-1L, and 1-MT (1-methyl-D-tryptophan) has opened a new door to immune activation against melanoma which can be considered for combinatorial therapy [65,66]. Based largely on data from anti-CTLA-4 treatment, reversal of immunologic suppression may have late but prolonged effects in both tumor response and survival presenting another challenge for the evaluation of combination therapy [67,68].

Melanoma cell lines often display constitutive up regulation of anti- apoptotic molecules such as Bcl-2, which may partly account for resistance to chemotherapy. Yet, the combination of DTIC plus oblimersen, anti-sense Bcl-2, had little effect on survival [69,70]. Hersey and Zhang [71] have proposed combining pro-apoptotic drugs, with immunotherapy. Their results suggest that within a polyclonal population sensitive cells have predominantly activated the FADD/caspase 8 pathway whereas resistant cells have dominant activation of NF-kB and MEK pathways. Thus, subsets of melanoma cells acquire resistance to apoptosis unless intrinsic anti-apoptotic signaling is interrupted. Up-regulation of the anti-apoptotic Bcl-2 family member Mcl-1 is another mechanism critical for protection of melanoma cells against endoplasmic reticulum stress-induced apoptosis [72]. Pro-apoptotic agents have not yet been fully exploited outside of chemotherapy combinations and may constitute an important group agent to combine with growth signaling inhibition and immunotherapy [73].

Additionally, some melanomas may be susceptible to treatment with anti-angiogenic agents. When anti-angiogenic agents are used in combination with chemotherapy, the response rate to chemotherapy may be improved [74]. Understanding the mechanisms of resistance to bevacizumab [an antibody that binds to and neutralizes the biologic activity of human vascular endothelial growth factor (VEGF)] may be relevant to its use in combination. It is possible that effects are limited by either intrinsic resistance to bevacizumab in an inflammatory tumor milieu or lack of activity to chemotherapy, so that other combinations with these agents need to be evaluated. In models, vascular disrupting agents that target the established tumor vasculature result in extensive intratumoral hypoxia and cell death. However, a rim of viable tumor tissue from which angiogenesis-dependent regrowth can occur, may depend on mobilization and tumor colonization of circulating endothelial progenitor cells (CEP). Thus co-treatment that blocks CEPs might not result in further tumor regression, but could affect the ability of tumors to continue growth after therapy. Low dose metronomic chemotherapy, such as cyclophosphamide 50 mg daily, has been proposed as an anti-angiogenic agent which may potentiate the effectiveness of vascular disrupting agents [75].

Another group of promising agents to consider for combination therapy are histone deacetylase (HDAC) and methylation inhibitors; preclinical reports have shown that HDAC inhibitors synergize with cytotoxic agents, such as DNA topoisomerase, imatinib, bortezomib, and various biologic agents. The same studies have shown that when combining agents, the sequence and doses may have a profound impact. As is the case for many biologic agents, the optimal doses for target inhibition may not directly correlate with the traditional maximum-tolerated dose (MTD). A phase I study suggested an impressive response rate in patients with otherwise refractory melanoma, breast, cervical, prostate, and small-cell lung cancer [76]. Other concepts not discussed here but which deserve mention are inhibitors of Notch1, Wnt, and HEDGEHOG pathways and cellular adhesion molecules [77-80].

This discussion is not meant to cover all emerging concepts for the treatment of melanoma, but provides examples of the myriad permutations that may need to be tested in an organized fashion to identify the best possible combinatorial approaches.

## Limitations of future trial design and potential solutions

Drugs such as the B-RAF inhibitors have shown efficacy in early clinical trials, and it is likely they will become standard for first line treatment in selected patients. Although these examples provide convincing proof of principle that some of these novel approaches constitute new tools for the treatment of cancer, they have also proven that a single drug is unlikely to induce lasting clinical benefit. New drugs are licensed primarily on the basis of single agent safety and efficacy for a specific clinical indication. Yet once licensed, active drugs such as bevacizumab, trastuzumab, and bortezomib have been available for new uses in combinations, typically in empirically based clinical trials. However, even this less than optimal process, applies only to drugs that are available because of their obvious effectiveness in early trials, which may eliminate many that are likely to work in selected patients or in combination. Thus, the future of therapy will depend on cooperation among various stakeholders and an organized effort to derive the maximum information from clinical trials. This also requires a willingness to share information and minimizing obstacles placed by intellectual property and related financial interests. It is reasonable to expect enlightened self-interest to recognize the need for multiple participants to focus on knowledge gained and the overall benefit of treating cancer patients in clinical trials. A paramount step along the way would be to foster arrangements that could allow more access to agents both for clinical and pre-clinical studies and more complete access and analysis of study results. For many patients, the molecular characterization of melanoma may allow predictive classification for selection of patients entering trials [81]. The ability to utilize effective agents in combination and/or in sequence could allow individualized treatment approaches adapted to a tumor's evolving biology [82]. Trials could be designed and made available for the patient rather than the patient made available for treatment. Thus, it is our hope that in the future appropriate combinations of drugs will be readily available to address the likely limits of single agents as has been successfully done with chemotherapy of some cancers and persistent infections.

## Competing interests

PAA participated to advisory board from Bristol Myers Squibb and GSK; He receives honoraria from Schering Plough and Genta

## Authors' contributions

All Authors: 1) made intellectual contributions and participated in the acquisition, analysis and interpretation of data; 2) have been involved in drafting the manuscript; and 3) have given final approval of the version to be published.
